# Effects of parent and child behaviours on overweight and obesity in infants and young children from disadvantaged backgrounds: systematic review with narrative synthesis

**DOI:** 10.1186/s12889-016-2801-y

**Published:** 2016-02-13

**Authors:** Catherine Georgina Russell, Sarah Taki, Rachel Laws, Leva Azadi, Karen J. Campbell, Rosalind Elliott, John Lynch, Kylie Ball, Rachael Taylor, Elizabeth Denney-Wilson

**Affiliations:** Faculty of Health, University of Technology, Sydney, NSW Australia; Centre for Physical Activity and Nutrition Research, Deakin University, 221 Burwood Highway, Burwood, VIC 3125 Australia; School of Population Health, University of Adelaide, Adelaide, SA Australia; University of Otago, Dunedin, New Zealand; Centre for Obesity Management and Prevention Research Excellence in Primary Health Care (COMPaREPHC), Sydney, Australia

**Keywords:** Obesity, Parents, Children, Socioeconomically disadvantaged, Indigenous, Eating, Food, Sedentary, Activity, Weight

## Abstract

**Background:**

Despite the crucial need to develop targeted and effective approaches for obesity prevention in children most at risk, the pathways explaining socioeconomic disparity in children’s obesity prevalence remain poorly understood.

**Methods:**

We conducted a systematic review of the literature that investigated causes of weight gain in children aged 0–5 years from socioeconomically disadvantaged or Indigenous backgrounds residing in OECD countries. Major electronic databases were searched from inception until December 2015. Key words identified studies addressing relationships between parenting, child eating, child physical activity or sedentary behaviour and child weight in disadvantaged samples.

**Results:**

A total of 32 articles met the inclusion criteria. The Mixed Methods Appraisal Tool quality rating for the studies ranged from 25 % (weak) to 100 % (strong). Studies predominantly reported on relationships between parenting and child weight (*n* = 21), or parenting and child eating (*n* = 12), with fewer (*n* = 8) investigating child eating and weight. Most evidence was from socio-economically disadvantaged ethnic minority groups in the USA. Clustering of diet, weight and feeding behaviours by socioeconomic indicators and ethnicity precluded identification of independent effects of each of these risk factors.

**Conclusions:**

This review has highlighted significant gaps in our mechanistic understanding of the relative importance of different aspects of parent and child behaviours in disadvantaged population groups.

**Electronic supplementary material:**

The online version of this article (doi:10.1186/s12889-016-2801-y) contains supplementary material, which is available to authorized users.

## Background

The rapid global increases in the prevalence of childhood overweight and obesity herald an urgent need to understand how to improve infant and early childhood risk for adiposity [1, 2]. Overweight in infancy is important given it is likely to track into childhood [3–5]and later life [6, 7]. Similarly, eating and activity habits and preferences appear to be learned in infancy and childhood [8–10], and may also track into adolescence and adulthood [11–13]. Given this persistence and the consequent effects of adiposity across the lifespan, focusing attention on infants and young children may have benefits in the longer term.

Children from socioeconomically disadvantaged backgrounds and Indigenous families carry the burden of overweight and obesity disproportionately [1, 14]. In Australia over one quarter (27 %) of children from disadvantaged backgrounds are overweight or obese compared to less than one fifth (19 %) of socioeconomically advantaged children [15]. Similar patterns are observed in the USA [16], France [17], the UK [18]and Canada [19]. Indigenous children are also at greater risk of overweight and obesity than their non-Indigenous peers. A recent study of urban Indigenous infants in Australia, for instance, reported that more than a third (36.9 %) of the children in that cohort were overweight or obese at two years of age, while nationally the figure is close to 20 % [20]. Understanding the most appropriate ways in which to prevent overweight and obesity in our disadvantaged groups is therefore of particular importance. Despite this, the evidence base upon which to design interventions remains poor, and there is some evidence that current obesity prevention programs may widen the disparity in obesity prevalence [21]. Further exploration of the determinants of socioeconomically patterned overweight and obesity in infancy and early childhood is required.

The reasons why children from disadvantaged backgrounds are at greater risk of becoming overweight are likely to be multifaceted, although the family environment must play a major role [22, 23]. Those aspects of the family environment that are both prevalent and amenable to intervention are of greatest interest, namely parenting and dietary and physical activity behaviours. Although a range of parental feeding behaviours, such as breastfeeding, formula feeding, timing and type of solid foods introduced, parental control in feeding, parental pressure to eat or restriction in feeding [24–26] have been linked with excessive weight gain in children [27–30], most existing research has focused on mixed populations or those of higher advantage [24]. Evaluating these behaviours in less advantaged populations appears to have been rarely examined. Similarly, although children’s eating and activity habits and preferences vary by socioeconomic factors [31–34], exactly how these behaviours explain the increased risk of obesity amongst those experiencing disadvantage is not well understood. The available literature from a range of OECD countries does point to similar predictors for these groups, with many of the behaviours associated with excess weight gain being more prevalent in these groups [35–40], suggesting common pathways.

In summary, although there are a number of plausible pathways through which disadvantaged children may experience greater weight gain, it is presently unclear as to how parental feeding, child or infant activity and sedentary behaviours each contribute. By conducting this review we aimed to synthesize research on potential pathways through which disadvantaged infants and children aged up to 5 years and from OECD countries may experience greater weight gain. In particular, we focused on the roles of (a) parenting behaviours, (b) children’s eating and (c) children’s physical activity or sedentary behaviour as mechanisms for linking socioeconomic disadvantage and Indigenous status to greater weight gain in these groups. This emphasis on mechanistic pathways is intended to illuminate the most promising avenues for future interventions.

## Methods

### Study selection criteria

**Fig. 1:**
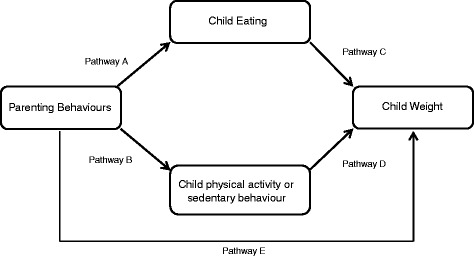
Studies were included in the review if they assessed any one or more of the selected pathways through which parents and children may affect children’s overweight or obesity

Studies were selected for inclusion in the review if they addressed one of the following pathways, nominated due to previously reported associations with overweight or obesity in a variety of populations (see Fig. 1): (A) between parenting behaviours and child eating, (B) between parenting behaviours and child activity (physical activity or sedentary behaviours), (C) between children’s eating and children’s weight, (D) between child activity levels (including sedentary behaviours and physical activity) and child weight and (E) between parenting behaviours and child weight. Child eating was defined as dietary intake (including breast milk or formula), diet patterns, intakes of specific foods or beverages, food choices, food preferences, eating styles and eating behaviours. The age at which children started consuming solid foods was also included as a ‘child eating’ variable [41, 42]. Parenting behaviours included specific feeding behaviours (e.g. using food as a reward, modelling) and general parenting behaviours. All studies assessing pathways C, D and E had to provide measures of anthropometric status.

To be included, studies needed to focus on low socio-economic or Indigenous groups or with the overall results stratified by socioeconomic or Indigenous group, or to report on interactions between socioeconomic or Indigenous group and the pathway variables. Socioeconomic disadvantage was defined based on families being described as having low income, low level of education or occupation, and/ or living in an area defined as disadvantaged using aggregate indicators. Indigenous was defined using Cobo’s criteria [43]. As our focus was on early life, only studies of children aged 0–5 years were included. Studies focusing on weight loss or with children with underlying medical conditions were excluded.

No limitations were placed on publication year, although studies needed to be published in English and use human participants. However, we limited our search to OECD countries as our focus was on the effects in high-income countries [44, 45]. Studies included had to be primary studies or papers presenting secondary data analyses from these studies, and be published in a peer-reviewed journal or edited book. The full search strategy is available from the authors.

### The search strategy

Between June 2013 and November 2014, we conducted a systematic literature search of ten electronic databases (Academic Search Complete, PsycINFO, CINAHL, Medline, EMBASE, Health Collection, Google Scholar, Joanna Briggs Institute, Scopus, Proquest), guided by the PRISMA statement [46]. The search terms child* OR infant* OR famil* OR parent* OR mother OR maternal OR father/Indigenous* OR socioeconom* OR socio-econom* OR poverty OR disadvant* OR unemploy*/obes* OR overweight OR weight/exercis* OR play OR activ* OR sedent* diet* OR nutrition OR Eating behavi* OR food/breastfe* OR “breast fe*” OR feeding OR Parent*/Empirical research OR clinical trial* OR randomi* OR qualitative OR cohort study OR quantitative were used to identify relevant literature. We also searched via subject headings in PsycINFO, CINAHL and Medline. Limiters were set to exclude literature reviews, systematic reviews, meta-analyses, letters, and editorials. We also visually scanned reference lists from relevant studies and undertook citation searching.

### The study selection process

Articles retrieved through the electronic search process were entered into an EndNote bibliographic database. A process of electronic elimination of duplicates subsequently took place. Titles and abstracts were then screened by two authors (CGR and ST) and papers were classified as either (A) appearing to meet the selection criteria, (B) meeting selection criteria difficult to determine, or (C) excluded articles (did not meet selection criteria or duplicate). Full document texts from the potentially eligible groups (A and B) were examined. Any discrepancies between the two authors were resolved by discussion. A third researcher (EDW) checked 10 % of the titles and abstracts classified as excluded studies, to check reliability of the screening process.

### Data extraction and synthesis

Two researchers (CGR and ST) extracted key data from each of the papers using a developed template. Extracted data covered bibliographic information, study background and aims, setting, inclusion and exclusion criteria, recruitment strategy, when and how data were collected, sample size and response rate, characteristics of parents (e.g. age, gender, education level, employment status, income, ethnicity, BMI), child/infant characteristics (e.g. age, gender, weight, whether breast- or bottle-fed), outcomes measured and relationships tested, covariates, definitions and measures of key variables, results, author’s interpretation and conclusions. In instances where publications reported multiple dependent variables only those results fitting the inclusion criteria were extracted. A cross check of 10 % of the extracted studies was undertaken by a third researcher (EDW) to ensure accuracy of data extraction. Differences in data interpretation and extraction were resolved through discussion.

### Quality assessment

The quality of the selected studies was appraised using the Mixed Methods Appraisal Tool (MMAT) [47] independently by one of the authors (RE), with 10 % of the sample cross-checked by another author (EDW). The MMAT was selected in order to accommodate the various study methodologies and to enable us to meet the aims of the review. The MMAT comprises two screening questions (applied to all studies) and four questions for five broad study methodologies; qualitative, quantitative randomized controlled trials, quantitative non-randomized controlled, quantitative descriptive and mixed methods. Mixed methods studies are evaluated using the appropriate criteria for the study methodologies employed in the particular study as well as the mixed methods criteria and the methodology with the lowest score is the final quality rating. A quality rating is derived from the number of ‘yes’ responses to the criteria; this is either reported as a raw score or expressed as a percentage of the total number of criteria for that study methodology. Quality ratings range from a raw score of zero to four, (0-100 %) where zero indicates that none of the criteria were met and four (100 %) indicates that all criteria were met. The MMAT has been extensively tested for reliability and validity [47]. A quality rating was expressed as a percentage and derived by dividing the number of ‘yes’ responses by the number of applicable criteria multiplied by 100. To ensure accuracy of the quality assessment of the sub-studies in our review the main study was also located and the protocol thoroughly examined.

## Results

**Fig. 2:**
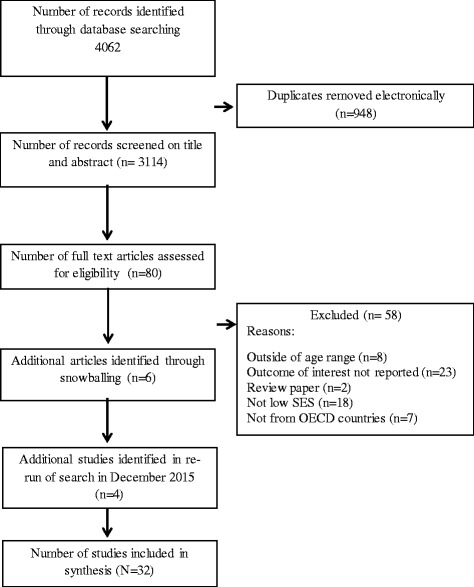
Flowchart depicting the search strategy and selection of articles for inclusion in the review on the effects of parents and children on overweight or obesity in disadvantaged children

The initial search process resulted in 4062 documents. Removal of duplicates resulted in 3114 articles, and the screening process reduced this further to 80. After analysing full document texts from the 80 potentially eligible articles, 22 studies remained. An additional 10 studies were included, six after searching reference lists and citation searching and four studies when the search was repeated. A total of 32 articles reporting on 31 studies were included in the review (Fig. 2).

### Study characteristics

The studies were heterogeneous in their design, context, focus and quality although the majority (16 of 32) of the studies used a cross-sectional self-reported survey to collect data from parents about themselves and their children. Longitudinal observational designs were also used (*n* = 6). Only one Randomised Controlled Trial (RCT) was included. The majority of the studies (*n* = 26) were conducted in the USA, with the remainder from various countries in Europe (*n* = 4) and Mexico (*n* = 1). A large proportion of the studies from the USA (*n* = 10) were of mixed or minority ethnic groups of low income. Two studies were of Indigenous populations of Native Americans and Native Alaskans from the USA and Canada, respectively. Other ethnicities were German (*n* = 1), Mexican (*n* = 3), Flemish (*n* = 1) and Dutch (*n* = 2). Three studies did not report ethnicity. Participants were typically recruited from primary care settings (e.g. Special Supplemental Nutrition Program for Women, Infants and Children, WIC, *n* = 19), preschools or kindergartens (*n* = 5) and community centres (*n* = 4). Just four studies reported using a theoretical model or conceptual framework to inform their study design. Articles were published between 2001 and 2014.

### Quality assessment

The quality rating for the studies ranged from 25 % to 100 %, with four articles rated at 25 %. Nine studies received a rating of 100 % with 13 rated at 75 %. Six further studies received a 50 % rating.

### Synthesis of findings

Key findings reported here reflect the pathways proposed in Fig. 1. A summary of each study is presented in Additional file 1: Table S1.

### Evidence for the association between parenting and child eating (Pathway A)

Twelve of the 32 studies examined associations between parenting and child eating (Pathway A, Fig. 1, Additional file 1: Table S1). The examined variables included parental feeding styles, parental capacity for resilience and an array of parental feeding practices such as control, pressure to eat and rewarding for eating. Seven studies [29, 44, 48–52] focused on various elements of dietary intake and one study [29] focused on dietary quality. Four studies [48–50, 53] examined associations between parenting practices and soft drink consumption; three [29, 49, 51] focused on fruit and vegetable consumption; and one on formula feeding [54].

#### Parental feeding practices

Several studies showed associations between maternal feeding practices and dietary outcomes in infants and children. Wijtzes et al. [50], in a high quality study, reported that parental modelling, ‘pressure to eat’, ‘restriction’ and ‘monitoring’ were predictive of Dutch children’s dietary intakes. Two other studies of moderate quality [29, 52] also reported cross-sectional associations between various parental feeding practices and children’s diets. However, null relationships between other parental feeding practices and infants’ or children’s dietary intakes were also reported in these two studies. Another high quality study [53] also noted that amongst Flemish mothers with lower levels of education parental modelling (avoiding negative modelling) was not associated with children’s soft drink consumption.

#### Availability and accessibility of food to children

Just one (high quality) study considered home food availability and accessibility reporting these constructs mediated associations between lower maternal education and Flemish children’s increased soft drink consumption [53].

#### Parental feeding style

Two high quality studies [53, 55] reported relationships between parental feeding style and children’s food or beverage intakes: De Coen [53] noted that Flemish parents’ permissive parental feeding style was associated with children’s increased soft drink consumption [53]. While Chaidez and Kaiser [55] reported that overweight or obese Latino mothers’ use of indulgent feeding was positively associated with their toddler’s intake of fat, saturated fat and sweetened beverages but not total energy six months later [55] and that maternal authoritative feeding style was predictive of toddlers’ lower consumption of sweetened beverages. Two other lower quality studies also reported positive associations between parental feeding styles and children’s food or beverage intakes, with parental feeding pressure, indulgent coaxing, restrictive feeding, authoritarian and uninvolved feeding styles being important [44, 51].

#### General parenting

Lim and colleagues [49] (quality rating 75 %) reported that low-income African American caregivers’ (*n* = 317) greater capacity for resilience was associated with children’s higher fruit and vegetables and lower soda intakes at baseline, and soft-drink consumption four years later.

### Evidence for the association between parent behaviour and child physical activity/sedentary behaviour (Pathway B)

Two studies examined associations between parenting and children’s physical activity or sedentary behaviour (Pathway B, Fig. 1) and both reported positive findings. In the higher quality study Wijtzes and colleagues [56] describe that parental (*n* = 268) modelling (maternal TV viewing) mediated almost a quarter of the effect of low maternal educational level as a predictor children’s TV viewing time. Dawson-McClureet al’s [57] lower quality (score 50 %) pre-post intervention with a low-income US sample of mixed ethnicity (76 % Afro-Caribbean) also reported associations between parenting behaviours and child physical activity and sedentary behaviour, but not weight.

### Evidence for the association between child eating and child weight (Pathway C)

#### Children’s dietary intake

Several studies examined relationships between dietary intake and body weight in a variety of disadvantaged groups [24, 58–63]. Layte et al’s high quality study [63] reported negative associations between diet quality and children’s weight at three years in a large (*n* = 11134) Irish study. Six papers tested associations between sweetened beverage consumption and weight [24, 58, 59, 61, 62, 64] with the only high (100 %) quality [58] study reporting associations between sugar-sweetened beverage (SSB) consumption and weight. Other studies reported that vegetable intake [64] and diet quality [59] were not associated with weight whilst consumption of high fat containing snacks was associated with greater obesity in Mexican children [61].

#### Children’s eating behaviours/appetitive traits

Just one, good quality (100 %), study examined associations between parental feeding and children’s eating behaviours or appetitive traits. Powers et al. [65] reported no relationship between the African American WIC mothers’ reports of the preschool children’s ‘food responsiveness’ or ‘desire to drink’ and child BMI z-score.

### Evidence for the association between child physical activity/sedentary behaviour and weight (Pathway D)

Two studies examined associations between physical activity or sedentary behaviour and children’s weight (Pathway D, Fig. 1) and both of these studies reported associations between the time spent watching TV and children’s weight. Layte and colleagues’ [63] longitudinal prospective cohort study (*n* = 790) was rated as high quality, whilst Dennison et al’s [33] cross-sectional study of WIC participants (*n* = 1761) was rated as good (75 %) quality and controlled for other variables such as the child’s age, sex and ethnicity.

### Evidence for the association between parental feeding practices or parenting behaviours and child weight (Pathway E)

There were 21 studies included in this review that examined the correlation between parenting behaviours and child weight. Variables considered were breastfeeding, formula feeding and an array of parental feeding behaviours such as pressure to eat and restriction.

#### Breastfeeding or formula feeding

Fifteen studies, of variable quality, investigated relationships between breastfeeding, formula feeding, bottle-feeding and weight. Thirteen of the studies were completed in the USA, ten of which collected data from WIC, including both studies examining milk bottle-feeding. Many of these studies reported a low prevalence of any breastfeeding or breastfeeding extending beyond 6 months of age [58, 63, 66, 67]. However, four good or high quality analyses demonstrated that breastfeeding appeared protective against future weight gain in non-Hispanic White children attending WIC clinics [66, 67], a mixed ethnicity sample [35] and in an Irish sample [63]. By contrast, breastfeeding did not appear to be related to later weight in non-Hispanic Blacks or Hispanic children [59, 64], and in another study of good quality no associations were reported between feeding mode and weight [68] in a predominantly White sample.

Two studies of moderate quality (quality score 75 %), with a predominantly (75 %) Hispanic WIC population [69] and the other with Mexican mothers [61] reported that formula feeding increased the risk of overweight. In the high quality study of Gibbs and Forste [35] which included a large (*n* = 1527) sample of mixed ethnicity reported a positive association between predominant formula feeding versus predominant breastfeeding and weight between the ages of 9 and 24 months, when controlling for several mother and child confounders. Bogen et al. [66] and Layte et al. [63] also demonstrated that the protective effect of breastfeeding was reduced with some formula use.

#### Bottle feeding

Two high quality studies [35, 58] reported associations between bottle-feeding practices and weight. May et al. [58] noted that bottle use at 18 months (which often contained SSBs) was predictive of child overweight in their small (*n* = 134) ethnically diverse sample, whilst Gibbs and Forste [35] reported that putting a child to bed with a bottle was associated with higher weight at 24 months.

Bonuck and colleagues published three studies [4, 70, 71] on bottle-feeding and weight in predominantly Hispanic samples recruited from WIC centres. Results were mixed, with bottle use being predictive of obesity but not overweight [4, 71], being predictive in toddlers but not pres-choolers [4] and not predicting weight or energy intake in 12–13 month olds [70]. These studies were rated as low-poor quality.

#### Age of introduction to solid foods

Four studies examined relationships between the age at which solid foods were introduced to infants and weight gain or dietary intakes. The two high quality studies reported associations between earlier introduction of solid foods and greater weight gain [35, 63]. In contrast, two other studies reported no associations between the age of introduction of solids and Mexican infants’ (*n* = 810) [61] or Black and Hispanic infants’ (*n* = 96) [69] weight.

#### Parental feeding practices

Eight of the included studies investigated relationships between parental feeding practices and weight in infants or children. Two higher quality studies noted an association between parental use of ‘pressure’ and infant weight. Thompson et al. [44] and Powers [65] reported that parental ‘pressuring’ was cross-sectionally and prospectively associated with lower infant weight in low-income African American samples (*n* = 217 and *n* = 296 respectively). This relationship was not observed by Worobey and colleagues [72] in their small, cross-sectional sample (*n* = 49) of low-income Hispanic mothers of four-year-old children (quality score 50 %). ‘Feeding restriction’ was also associated with higher BMI in some [44, 73] but not all [72] studies.

Starling Washington et al. [32] noted (quality 75 %) variable relationships between the measured feeding practices and chid weight whereas Hurley et al. [73] (*n* = 297) and Faith et al. [24] (*n* = 1797), reported no differences in feeding practices and child weight (quality 25 % and 50 % respectively).

These relationships may differ according to parental weight status: In Powers’ high quality study [65] in which several child- and parent-related confounders were controlled for, greater maternal ‘restriction’ and ‘control’ were both associated with higher child BMI z-score in obese mothers only. In non-obese mothers maternal ‘restriction’ was associated with lower child BMI z-score whereas ‘control’ was not related to child BMI z-score in non-obese mothers. Murashima et al. [52] reported cross-sectional associations between parents’ use of ‘control’ and ‘contingency’ and children’s higher BMI scores (quality 75 %). Likewise, another study [60] found that when controlling for the child’s age, only one of the thirteen measured parental feeding behaviours addressing maternal control of child eating (‘child takes food from refrigerator/pantry between meals’) was associated with obesity and none were associated with overweight.

#### Parental feeding styles

Associations between parental feeding style and infant or child weight were examined in two studies. Chaidez and Kaiser’s high quality study [55] found that indulgent and authoritative parental feeding styles did not cross-sectionally associate with toddler weight in a Latino sample (*n* = 94). Hughes et al. [74] reported that indulgent and authoritarian, but not authoritative or uninvolved parental feeding were associated with BMI in preschool children (quality 25 %).

#### General parenting

Two good quality studies examined the effects of general parenting measures on weight-related outcomes in children. Lim and colleagues [49] reported that higher caregiver resilience was associated with a lower relative risk of children remaining overweight or obese versus normal weight over the four years of the study, but did not predict whether a child transitioned from normal weight to overweight or obese. Starling Washington et al. [32] found that scores on ‘response to distress’ (higher in normal weight group) and ‘cognitive growth fostering’ (higher in obese group) were significantly different between the two groups at enrolment, but only ‘cognitive growth fostering’ was different six months later. None of the scores on the measure of the proximal and distal home environments were significantly different between the two groups.

## Discussion

This review examined the evidence for effects of parental and child behaviours on overweight and obesity in disadvantaged groups. Overall, in disadvantaged (and particularly, Indigenous) populations, there remains a relatively scant body of evidence describing the influence on children’s weight of most of the behavioural variables assessed; and that where more evidence exists (e.g. for some categories like parental feeding practices) the variation in study designs, dietary outcomes, measures and other limitations make it difficult to draw strong conclusions. The review has shown that further exploration of causal pathways linking parenting and children’s eating and activity levels with weight in a range of disadvantaged groups is needed.

### Associations between parenting behaviours and child eating and weight

Findings on associations between parenting and child or infant weight and dietary intakes were generally suggestive of maternal feeding behaviours being in reaction to children’s weight status [44, 65]. Parents with heavier children used more restriction [72, 73] and less pressure to eat [44, 65, 72] reflecting findings in samples of mixed SEP and ethnicity [24, 75–78]. Parental feeding pressure and restriction were also associated with children’s greater intakes of unhealthy foods and beverages in some [29, 79], but not all [44] of the included studies, as well as greater energy intake [44]. Finally, lower responsive feeding scores were associated with toddler overweight [73].

### Associations between child eating and weight

Of the included studies assessing the importance of children’s diet, there was evidence that consumption of soft drinks [24, 62], was associated with greater weight [32]. Juice consumption was also predictive of greater weight in some [24, 32, 60] studies. There were several contrasting findings, with obese children eating more fruit than non-obese children (this study did not report on vegetable consumption) in one study [32] whilst another [59] found that neither fruit nor vegetable intake was related to overweight. Similarly, obese children ate more bread and other carbohydrates as well as total calories in comparison to non-obese children in one study [32] but in another [60] grain intake was not linked with weight status. In another study [59] the measures of diet were related to infant overweight.

Dietary intake (foods and beverages) is associated with overweight and obesity in samples of mixed ethnicity and SEP [80, 81] although results are not always consistent [82–84]. It is possible that differences in children’s initial risk of obesity may account for some of the observed discrepancies across studies [24, 62]. Further, a number of the included studies recruited participants from WIC or other clinics where participants would be receiving information on nutrition, weight and health, which may have affected their behaviours or responses [24, 60]. Parental education level is also found to vary widely, even within the low-income WIC populations, for instance [65, 72] and may have confounded results.

### Associations between breast- and bottle-feeding and child weight

Results on associations between breastfeeding and weight were mixed. Of the six studies reporting on the relationship between breastfeeding and weight status, only two reported an inverse dose–response relationship between breastfeeding and infant overweight [66, 85] with a minimum duration of three or four months of breastfeeding required. Importantly, these studies had a very large sample size, a long duration of follow-up and were rated high in quality, potentially allowing for modest protective effects of breastfeeding to be detected than in the other smaller studies. However, the positive effect of breastfeeding found in both of these studies was only seen in non-Hispanic Whites and no other racial or ethnic group. This is significant given that in two of the studies reporting no relationship between breastfeeding and overweight [59, 61] the study populations were entirely Hispanic; and in one of the other studies also finding no protective effect, [58] just over half of the participants were Hispanic.

The reported effects of breastfeeding on infant weight were often sizeably reduced when a range of other variables were controlled for. For instance controlling for variables previously linked with child weight such as maternal smoking, weight gain during pregnancy or maternal obesity substantially weakened or negated the effects of breastfeeding on overweight or obesity [66–68]. Moreover, breastfeeding has been associated with other health behaviours such as a more positive eating pattern and later introduction of solid foods [86]; behaviours also independently associated with obesity in childhood [35]. It is possible that the reported associations between breastfeeding and infant and child overweight seen in disadvantaged families could therefore at least be partly accounted for by other health behaviours associated with greater breastfeeding duration, several of which were not controlled for in the present studies[87, 88]. A further complication was that only one study [66] examined differences between exclusive breastfeeding and breastfeeding with concurrent formula feeding ([59] measured it but did not report results) despite suggestions that a large proportion of low SES mothers breast- and formula- feed concurrently [69].

It is unclear why the protective effect of breastfeeding in relation to weight status is found almost exclusively in populations of White European descent [89]. Ethnic differences in the effects of breastfeeding on weight suggest a behavioural, rather than a biological mechanism. There is evidence, for example, that Hispanic, Black and White mothers feed their children differently in terms of breastfeeding, feeding children SSBs, but also restriction and indulgent feeding, for instance [90, 91]. Other possible explanations are residual confounding [92], biological or socioeconomic factors [60]. Furthermore, small numbers of breastfeeding mothers in non-White groups, particularly after six months, mean that studies of breastfeeding in such groups may be underpowered. Nonetheless, studies of socioeconomically heterogeneous groups of children have shown that breastfeeding – initiation, longer duration or exclusivity – may exert a modest protective effect on child overweight [89, 93, 94]. The reported associations between formula feeding and weight were also mixed, likely because formula feeding was rarely examined and when it was definitions and measures of formula feeding varied [35, 54, 58, 61, 66, 69]. Despite this, formula feeding appeared to reduce the protective effects of breastfeeding on weight gain, whilst frequency of formula feeds was somewhat predictive of overweight [69, 95].

Diversity in measures and definitions of formula feeding behaviours and indeed breastfeeding (e.g. whether ever breastfed, breastfed for six months or more, exclusively breastfed and so on) reported in these studies is a limitation. How mothers breastfeed, formula feed or feed from a bottle may be more important than simply whether mothers breast- or formula-feed [69, 96]. The reasons for this may be that satiety responsiveness or calorific self-regulation, as well as maternal sensitivity to infant hunger and satiety cues are important mediators through which maternal feeding practices may influence weight gain [69, 97]. However, it is difficult to determine whether these potential behavioural mediators existed in the other studies, as just one of the studies included in this review assessed these factors [69]. In order to understand the relative importance of breastfeeding and formula feeding as predictors of overweight in disadvantaged infants and children higher quality studies are needed that use well-defined and detailed measurements of breastfeeding and formula- behaviours, including protein content and type, methods of preparation and amounts consumed, and the introduction and provision of solids.

### Associations between age of introduction of solid foods and child weight

The age of introduction to solid foods was also rarely assessed, with only four studies examining this as a predictor of infant adiposity [35, 61, 63, 69]. Just one of these studies, the highest in quality, reported significant associations [35]. In socioeconomically diverse populations, introduction of solid foods before an infant is four months of age has been associated with greater weight gain [98]. However, as noted earlier, early introduction of solid foods is also associated with other behaviours linked to obesity including earlier introduction of high fat foods and SSBs [35, 61, 86]. As these obesity-promoting behaviours cluster and are more prevalent in disadvantaged families, isolating the independent impact of early solids introduction on weight gain was challenging within the available set of studies.

### Associations between physical activity or sedentary behaviour and child weight

There was only one study [33] examining associations between sedentary behaviour or physical activity and children’s weight and both reported significant positive results. The number of hours that children watched TV appeared to be an important correlate of excess weight [33]. Children from disadvantaged backgrounds watch more hours of TV per day than children from more advantaged backgrounds [99–101], however this relationship may be complicated by the influence of other demographic factors such as ethnicity [33]. It is also possible that greater TV viewing by low SES children is more strongly associated with obesity in these children because TV viewing is associated with other obesity-promoting behaviours, such as consumption of energy-dense foods [102]. However, due to the small number of studies included in the review examining the influence of sedentary behaviours or physical activity in disadvantaged groups, more research is required before any firm conclusions can be drawn.

Documenting the mechanisms that may explain the development of obesity-promoting habits amongst parents and young children from low socioeconomic or Indigenous backgrounds is fundamental to understanding how to prevent them. However a large number of the studies included in this review were of cross-sectional design, therefore limiting their ability to examine causality, including bi-directional effects. Bi-directionality is imperative to understand in parent–child interactions as parenting is at least partially reactive to the child’s extant characteristics such as weight status, temperament and eating behaviours [103, 104]. Moreover, because cross-sectional studies are unable to distinguish between incidence and persistence of overweight in children, testing associations between, for instance, dietary intake and weight in a sample that includes children who are already overweight is difficult. Prospective studies utilising validated and culturally reliable measures of key variables (e.g. parent feeding behaviours) are needed if we are to further reveal causal relationships in parent–child feeding (and activity) relationships in disadvantaged families. Additionally, future studies utilising large population-based samples in socioeconomically and ethnically diverse groups, measuring the possible pathways of effect and testing for mediators, whilst controlling for confounders, including mediator-outcome confounding, are needed.

This review has also highlighted that research in this area is hindered by the availability of appropriate or adequate measurement tools, a challenge that has been highlighted previously [74, 105, 106]. Many of the tools utilised in the included studies were either purpose-developed with few data on validity and reliability [48, 64], or were established tools that were developed in different ethnic and socio-economic groups (primarily high advantage White families) than in which they were applied [65, 107]. The appropriateness of these tools to collect data in disadvantaged ethnic minorities or Indigenous populations is unknown. Children in White, higher income families likely have very different feeding environments (e.g. foods available, parental feeding behaviours and beliefs) and both the types of relevant behaviours and their measurement may not be appropriate for other groups. Indeed the CFQ [75], a widely used tool to measure parental feeding behaviours [108] appears better able to detect associations between parental feeding and child weight in all-White mother-daughter samples [109, 110] than in other samples [111, 112]. Similarly, clear definitions of each of the concepts (e.g. early introduction of solids, breastfed, restriction) under study were often lacking and appeared to differ across studies. Future research aiming to develop and apply tools more specific to the target populations, different food groups and eating contexts may help tease out relationships.

Obesity prevention interventions may be less effective in disadvantaged populations [113], likely because they have not been tailored towards the specific requirements of disadvantaged families. The present review has highlighted that there is only a small evidence base explaining causal relationships between parent and child behaviours and children’s weight status upon which interventions tailored to disadvantaged groups could be designed. Overall, only a small number of factors that could affect weight gain in disadvantaged families has been considered. The focus to date has been on the duration of breastfeeding, socio-demographics, dietary intakes and a selected few parental feeding behaviours such as restriction, control and pressure to eat [108, 114]. Consideration of other factors that affect weight such as other parental feeding behaviours, or how children are breast- or formula-fed (e.g. feeding to appetite) has seldom been undertaken in disadvantaged groups. Additionally, because many of the parent and child behaviours associated with overweight co-occur [115], studies that isolate or control for confounding are needed if we are to elucidate mechanisms of effect.

There are a number of limitations to this review. The search strategy was limited to parental feeding, child eating, physical activity and weight although there are other factors that influence children’s weight trajectories. We used a limited number of search engines, did not examine grey literature, and limited our search to English language and therefore may have missed some relevant studies. We did not examine all possible relationships that may affect infant or child weight, including relationships between child sedentary behaviour and child eating. Furthermore, we examined only two disadvantaged groups– low income and Indigenous and restricted our geographic search to the OECD. Finally, we were unable to perform a meta-analysis due to heterogeneity in population types, measures and outcomes.

## Conclusions

Although children from disadvantaged families are at greater risk of overweight and obesity, the evidence base outlining reasons for this remain underdeveloped. The 32 articles included in this review examined parental feeding behaviours, children’s diets, breastfeeding, formula feeding, bottle use, age of introduction of solid foods and time spent in sedentary behaviour as risk factors for infant or child overweight and obesity. However, the measured predictors of children’s eating, activity, sedentary behaviour and weight differed considerably across studies. Therefore, in disadvantaged (and particularly, Indigenous) populations, evidence attesting to the influence on children’s weight of most of the behavioural variables assessed is scarce; and few studies measured the same combinations of predictors, confounders, mediators and outcomes. Research to enable greater understanding of the predictors of weight gain in disadvantaged populations remains essential if we are to design targeted and effective obesity prevention interventions.

## Additional file

Additional file 1: Table S1. Summary of the articles included in the review of the effects of parents and children on overweight or obesity in disadvantaged children. (DOCX 99 kb)

## Abbreviations

BMI: Body mass index
OECD: Organization for economic cooperation and development
MMAT: Mixed methods appraisal tool
RCT: Randomised controlled trial
WIC: Special supplemental nutrition program for women, infants and children
SSB: Sugar-sweetened beverage
