# Supplementary material for: Effects of parent and child behaviours on overweight and obesity in infants and young children from disadvantaged backgrounds: systematic review with narrative synthesis
Source: BMC Public Health. 2016 Feb 13;16:151. doi: 10.1186/s12889-016-2801-y (PMC4753044; doi:10.1186/s12889-016-2801-y)
Supplement: Additional file 1: Table S1. — Summary of the articles included in the review of the effects of parents and children on overweight or obesity in disadvantaged children. (DOCX 99 kb) [file 12889_2016_2801_MOESM1_ESM.docx]

**Table 1 – Summary of the articles included in the review of the effects of parents and children on overweight or obesity in disadvantaged children**

| **Authors and country** | **Indicator of disadvantage** | **Parent Ethnicity** | **Target group characteristics** | | **Sample size (disadvantaged group only)** | **Study design** | **Recruited from** | **^[[1]](#footnote-1)^Theoretical basis** | **Confounders identified/adjusted** | | **^[[2]](#footnote-2)^Results by pathway** | | | | | **^[[3]](#footnote-3)^Quality (%)** |
| --- | --- | --- | --- | --- | --- | --- | --- | --- | --- | --- | --- | --- | --- | --- | --- | --- |
|  |  |  | **Parent** | **Child** |  |  |  |  | **Parent** | **Child** | **A (parenting** 🡺 **child eating)** | **B (parenting** 🡺 **activity)** | **C (eating** 🡺 **weight)** | **D (activity** 🡺 **weight)** | **E (parenting** 🡺 **weight)** |  |
| Kavanagh et al. (2008), USA | ^[[4]](#footnote-4)^WIC eligibility | Not reported | Formula feeding caregivers | <4 months old | 61 | ^[[5]](#footnote-5)^RCT | Primary Care (WIC) |  | Not reported | Formula intake, | Parenting practices (overfeeding) 🡺 formula feeding (+) |  |  |  | Overfeeding Formula feeding 🡺 weight (+) | 25 |
|  |  |  |  |  |  |  |  |  |  | Baseline anthropometry |  |  |  |  |  |  |
| Lim et al (2011), USA | Areas with high proportion of residents <200% poverty level | African-American | Low income African-American caregivers | 3-6 years | 317 | Longitudinal cohort | Longitudinal cohort study focusing on the oral health of low-income African-American children and caregivers |  | Age, education | Baseline obesity | Resilience 🡺 Fruit and vegetable consumption (+) (baseline only) |  |  |  | Resilience 🡺risk of staying overweight or obese (-) | 75 |
|  |  |  |  |  |  |  |  |  |  |  |  |  |  |  |  |  |
|  |  |  |  |  |  |  |  |  |  |  | Resilience 🡺 soft drink consumption (-) |  |  |  | Resilience 🡺risk of staying transitioning from normal weight to overweight or obese (ns) |  |
| Chaidez and Kaiser (2011), USA | WIC eligibility | Latino | Latino mothers living in urban, suburban, and semi-rural communities | 12 - 24 months | 94 | Longitudinal cohort | Primary Care (WIC), various community facilities |  | Maternal weight, birthplace, income, height |  | Indulgent feeding 🡺 energy consumption (+) |  |  |  | Indulgent/authoritative feeding 🡺 weight (ns) | 100 |
|  |  |  |  |  |  |  |  |  |  |  |  |  |  |  |  |  |
|  |  |  |  |  |  |  |  |  |  |  | Indulgent feeding 🡺 sweetened beverages (+) |  |  |  |  |  |
|  |  |  |  |  |  |  |  |  |  |  |  |  |  |  |  |  |
|  |  |  |  |  |  |  |  |  |  |  | Authoritative feeding 🡺 sweetened beverages (-) |  |  |  |  |  |
| Powers et al (2006), USA | WIC eligibility | African-American | African-American mothers | 24-59 months | 296 | Cross-sectional | Primary Care (WIC) |  | Maternal age, education, income, employment, marital status | Age, sex |  |  | Food responsiveness 🡺 weight (ns) |  | Pressure to eat 🡺 weight (+) | 100 |
|  |  |  |  |  |  |  |  |  |  |  |  |  |  |  |  |  |
|  |  |  |  |  |  |  |  |  |  |  |  |  | Desire to drink 🡺weight (ns) |  | Restriction 🡺 weight (+ obese mothers only) |  |
|  |  |  |  |  |  |  |  |  |  |  |  |  |  |  |  |  |
|  |  |  |  |  |  |  |  |  |  |  |  |  |  |  | Control 🡺 weight (+ obese mothers only) |  |
| Worobey et al (2009), USA | WIC eligibility | Black (24%), Hispanic (76%) | Low income, minority bottle-feeding mothers | Infants | 96 | Longitudinal cohort | Primary Care (WIC) |  | Ethnicity, pre-pregnancy BMI, weight gain in pregnancy, age, parity |  |  |  |  |  | Frequency of formula feeds🡺 weight at 6 months (ns) | 50 |
|  |  |  |  |  |  |  |  |  |  |  |  |  |  |  |  |  |
|  |  |  |  |  |  |  |  |  |  |  |  |  |  |  | Frequency of formula feeds🡺 weight at 12 months (+)  Age of introduction of solids 🡺 infant weight at (ns) |  |
|  |  |  |  |  |  |  |  |  |  |  |  |  |  |  |  |  |
| Baughcum et al (2001), USA | WIC eligibility | White (72%), Non-white (28%) | Biological parents | 11-24 months | 285 (STUDY 1) | Cross-sectional | Primary Care (WIC) |  |  |  |  |  |  |  | Breastfeeding duration 🡺 weight (ns) | 75 |
|  |  |  |  |  |  |  |  |  |  |  |  |  |  |  |  |  |
|  |  |  |  |  |  |  |  |  |  |  |  |  |  |  |  |  |
| May et al (2002), USA | WIC eligibility | Hispanic (53%), White non-Hispanic (36%) Black, Asian or Native American (11%) | Parents | Overweight children 8 to 36 months | 134 | Longitudinal cohort | Primary Care (WIC) |  | Size of household | Baseline weight, sex, breastfeeding |  |  | ^[[6]](#footnote-6)^SSB intake 🡺 weight (+) |  | Breastfeeding versus formula feeding 🡺 weight (+) | 100 |
| Bonuck and Kahn (2002), USA | WIC eligibility | Hispanic (70%), African-American (23%), Asian (7%) | Parents | 18-56 months | 95 | Cross-sectional | Primary care (WIC) |  |  |  |  |  |  |  | Bottle-feeding (milk/sweetened beverage) 🡺 obesity (+) | 50 |
|  |  |  |  |  |  |  |  |  |  |  |  |  |  |  |  |  |
|  |  |  |  |  |  |  |  |  |  |  |  |  |  |  | Bottle-feeding (milk/sweetened beverage) 🡺 overweight (ns) |  |
| Bonuck et al (2010), USA | WIC eligibility | Hispanic (50%), Non-Hispanic Black (31%), Asian/Pacific islander (6%); White (non-Hispanic) (6%), other (7%) | Parents | Bottle fed (milk) children 12-60 months old | 150 | Cross-sectional | Primary Care (WIC) |  |  |  |  |  |  |  | Bottle-fed (milk) 🡺 weight (12-36 months olds) (+) | 50 |
|  |  |  |  |  |  |  |  |  |  |  |  |  |  |  |  |  |
|  |  |  |  |  |  |  |  |  |  |  |  |  |  |  | Bottle-fed (milk) 🡺 weight (37-60 months olds only) (ns) |  |
| Bogen et al (2004), USA | WIC eligibility | White (72), Black (28) | Low-income mothers | 0-4 years | 73458 | Retrospective cohort | Primary Care (WIC) |  | Race | Race |  |  |  |  | Exclusive breastfeeding 🡺 weight (- Whites only)  Breastfeeding with concurrent formula🡺 weight (-) (Whites only | 75 |
|  |  |  |  |  |  |  |  |  |  |  |  |  |  |  |  |  |
|  |  |  |  |  |  |  |  |  |  |  |  |  |  |  | ) |  |
| Grummer-Strawn et al (2004), USA | WIC eligibility | White, non-Hispanic (52%, / 42%, Black, non-Hispanic (24%)/ (29%), Hispanic (26%) /(14%), Other (8%)/ (5%) | Low income families | Born between 1988 and 1992 | 12 587 | Two longitudinal cohort data linkage analyses | Primary Care (WIC & Maternal and Child Health Block Grant) |  | Maternal age, education, pre-pregnancy BMI, weight gain in pregnancy, postpartum smoking | Sex, race, birth weight |  |  |  |  | Breastfeeding 🡺 weight (- Whites only)  Breastfeeding 🡺 weight (ns Hispanics & Blacks) | 100 |
|  |  |  |  |  |  |  |  |  |  |  |  |  |  |  |  |  |
|  |  |  |  |  |  |  |  |  |  |  |  |  |  |  |  |  |
| Watt et al (2013), USA | Attend clinic in low income area | Hispanic | Pregnant women or women with 2, 6 or 12 month old infants | 0-12 months | 152 | Cross-sectional | Primary Care clinic for perinatal care serving low-income predominantly Hispanic population |  | Language, marital status, size of household, income, insurance, WIC, obesity, pre-existing thyroid, diabetes, heart disease, depression |  |  |  | Dietary intake (SSB, sweets, fruit/veg juice, French fries) 🡺 weight (ns) |  | Breastfeeding 🡺 weight (ns) | 75 |
| Dennison et al (2002), USA | WIC eligibility | White (35%), Hispanic (33%), Black (23%) | Parents | 1-4 years | 2761 | Cross-sectional | Primary Care (WIC) |  |  |  |  |  |  | Hours of TV viewing per day 🡺weight (+) |  | 75 |
|  |  |  |  |  |  |  |  |  |  |  |  |  |  |  |  |  |
|  |  |  |  |  |  |  |  |  |  |  |  |  |  | TV in bedroom 🡺 weight (+) |  |  |
| Melgar-Quinonez et al (2004), USA | Attending Head Start, Healthy Start, WIC, county day care centres or migrant education programs | Mexican American | Latino, Mexican-American, Mexican, or Hispanic parents | Preschool aged with both parents at home | 204 | Cross-sectional | Community (Head Start; Healthy Start; WIC), County Day Care Centres, Migrant Education Programs |  |  | Age |  |  | Juice intake as proportion of daily energy 🡺 weight (+) |  | Child takes food from refrigerator/pantry between meals 🡺 weight (-) | 75 |
|  |  |  |  |  |  |  |  |  |  |  |  |  |  |  |  |  |
|  |  |  |  |  |  |  |  |  |  |  |  |  | Vegetable intake as proportion of daily energy 🡺 weight (ns) |  | Feeding practices (Less control) 🡺 weight (+) |  |
|  |  |  |  |  |  |  |  |  |  |  |  |  |  |  |  |  |
|  |  |  |  |  |  |  |  |  |  |  |  |  |  |  | Breastfeeding 🡺 weight (ns) |  |
| Worobey et al (2013), USA | WIC eligibility | Not reported | Low income minority mothers | Four year olds | 49 | Cross-sectional | Preschool and a study on nutrition and growth and enrolled in WIC |  |  |  |  |  |  |  | Restriction 🡺 weight (ns) | 75 |
|  |  |  |  |  |  |  |  |  |  |  |  |  |  |  |  |  |
|  |  |  |  |  |  |  |  |  |  |  |  |  |  |  | Pressure to eat 🡺 weight (ns) |  |
| De Coen (2012), Belgium | Mother's education level | Flemish | Parents | 2.5-7 years | 1561 | Cross-sectional | Schools |  | Age | Age, gender | Accessibility 🡺 Soft drink (+) |  |  |  |  | 100 |
|  |  |  |  |  |  |  |  |  |  |  |  |  |  |  |  |  |
|  |  |  |  |  |  |  |  |  |  |  | Permissiveness🡺 Soft drink (+) |  |  |  |  |  |
|  |  |  |  |  |  |  |  |  |  |  |  |  |  |  |  |  |
|  |  |  |  |  |  |  |  |  |  |  | Availability 🡺 soft drink (+) |  |  |  |  |  |
|  |  |  |  |  |  |  |  |  |  |  |  |  |  |  |  |  |
|  |  |  |  |  |  |  |  |  |  |  | Discouragement through rationale 🡺 soft drink (ns) |  |  |  |  |  |
|  |  |  |  |  |  |  |  |  |  |  |  |  |  |  |  |  |
|  |  |  |  |  |  |  |  |  |  |  | Avoiding negative modelling 🡺 soft drink (ns) |  |  |  |  |  |
| Starling Washington et al (2010), USA | WIC eligibility | Mexican American | Mexican American mothers of children (half overweight, half normal weight) | 2-5 years | 200 | Cross-sectional with 6 month follow up. | Primary Care (WIC) | ✓ |  |  |  |  |  |  | Someone sits with child at meals T1 🡺 Obesity (+) | 75 |
|  |  |  |  |  |  |  |  |  |  |  |  |  |  |  |  |  |
|  |  |  |  |  |  |  |  |  |  |  |  |  |  |  | Someone sits with child at meals T2 🡺 Obesity (ns) |  |
|  |  |  |  |  |  |  |  |  |  |  |  |  |  |  |  |  |
|  |  |  |  |  |  |  |  |  |  |  |  |  |  |  | Mother sits with child at meals T1 🡺 Obesity (+) |  |
|  |  |  |  |  |  |  |  |  |  |  |  |  |  |  |  |  |
|  |  |  |  |  |  |  |  |  |  |  |  |  |  |  | Mother sits with child at meals T2 🡺 Obesity (ns) |  |
|  |  |  |  |  |  |  |  |  |  |  |  |  |  |  |  |  |
|  |  |  |  |  |  |  |  |  |  |  |  |  |  |  | Who feeds child most meals T1 🡺 Obesity (ns) |  |
|  |  |  |  |  |  |  |  |  |  |  |  |  |  |  |  |  |
|  |  |  |  |  |  |  |  |  |  |  |  |  |  |  | Who else feeds child most meals 🡺 Obesity (ns) |  |
|  |  |  |  |  |  |  |  |  |  |  |  |  |  |  |  |  |
|  |  |  |  |  |  |  |  |  |  |  |  |  |  |  | Response to distress T1 🡺 Obesity (-) |  |
|  |  |  |  |  |  |  |  |  |  |  |  |  |  |  |  |  |
|  |  |  |  |  |  |  |  |  |  |  |  |  |  |  | Response to distress T2 🡺 Obesity (ns) |  |
|  |  |  |  |  |  |  |  |  |  |  |  |  |  |  |  |  |
|  |  |  |  |  |  |  |  |  |  |  |  |  |  |  | Cognitive growth fostering T1 & T2 🡺 Obesity (+) |  |
|  |  |  |  |  |  |  |  |  |  |  |  |  |  |  |  |  |
|  |  |  |  |  |  |  |  |  |  |  |  |  |  |  | Home Environment 🡺 Obesity (ns) |  |
| Wijtzes et al (2013), The Netherlands | Maternal education level | Dutch | Mothers | 48 months | 268 low (2814 total) | Prospective cohort (Generation R study) | Community |  | Maternal education | Sex and age | Monitoring 🡺 high fat snacks (-) |  |  |  |  | 100 |
|  |  |  |  |  |  |  |  |  |  |  |  |  |  |  |  |  |
|  |  |  |  |  |  |  |  |  |  |  | Restriction 🡺 high fat snacks (+) |  |  |  |  |  |
|  |  |  |  |  |  |  |  |  |  |  |  |  |  |  |  |  |
|  |  |  |  |  |  |  |  |  |  |  | Pressure to eat 🡺 high fat snacks (-) |  |  |  |  |  |
|  |  |  |  |  |  |  |  |  |  |  |  |  |  |  |  |  |
|  |  |  |  |  |  |  |  |  |  |  | Parental consumption of SSBs 🡺 high fat snacks (-) |  |  |  |  |  |
|  |  |  |  |  |  |  |  |  |  |  |  |  |  |  |  |  |
|  |  |  |  |  |  |  |  |  |  |  | Monitoring 🡺 SSBs (+) |  |  |  |  |  |
|  |  |  |  |  |  |  |  |  |  |  |  |  |  |  |  |  |
|  |  |  |  |  |  |  |  |  |  |  | Restriction 🡺 SSBs (ns) |  |  |  |  |  |
|  |  |  |  |  |  |  |  |  |  |  |  |  |  |  |  |  |
|  |  |  |  |  |  |  |  |  |  |  | Pressure to eat 🡺 SSBs (ns) |  |  |  |  |  |
|  |  |  |  |  |  |  |  |  |  |  |  |  |  |  |  |  |
|  |  |  |  |  |  |  |  |  |  |  | Parental consumption of SSBs 🡺 SSBs (-) |  |  |  |  |  |
|  |  |  |  |  |  |  |  |  |  |  |  |  |  |  |  |  |
|  |  |  |  |  |  |  |  |  |  |  |  |  |  |  |  |  |
|  |  |  |  |  |  |  |  |  |  |  |  |  |  |  |  |  |
| Wijtzes et al (2012), Netherlands | Maternal education level | Dutch | Pregnant women | 47-50 months | 2786 | Population-based prospective cohort study (Generation R study) | Community |  | Maternal education | Sex and age |  | Maternal TV viewing (Parent modelling) 🡺 child TV viewing (+) |  |  |  | 100 |
|  |  |  |  |  |  |  |  |  |  |  |  |  |  |  |  |  |
| Kroller & Warschburger (2008), Germany | Composite measure of family's net income and mother's education level | German | Mothers in low SES areas | 3-6 years | 219 | Cross-sectional | In-patient clinics and kindergartens |  |  |  | Pressure to eat 🡺 dietary quality (-) |  |  |  |  | 75 |
|  |  |  |  |  |  |  |  |  |  |  |  |  |  |  |  |  |
|  |  |  |  |  |  |  |  |  |  |  | Rewarding 🡺 fruit and veg (-) |  |  |  |  |  |
|  |  |  |  |  |  |  |  |  |  |  |  |  |  |  |  |  |
|  |  |  |  |  |  |  |  |  |  |  | Child’s own control 🡺 fruit and veg (-) |  |  |  |  |  |
| Jimenez-Cruz et al (2010), Mexico | Eligibility for government run primary health care program | Mexican | Mothers | Healthy children aged 5-24 months | 810 | Cross-sectional | Primary Care (local vaccination centres) |  | Household income |  | Age of solids intro 🡺 weight (ns) |  | Consumption of high fat snacks 🡺 weight (+)  Consumption of soft drink 🡺 weight (+)  Consumption of juice 🡺 weight (ns) |  | Formula feeding / formula + breastfeeding 🡺weight (+)  Exclusive breastfeeding 🡺 weight (-) | 75 |
| Thompson et al (2013) USA | WIC eligibility | African-American | First-time African-American mothers aged 18 to 35 years | 3-18 months | 217 | Longitudinal observational cohort | Primary Care (WIC) | ✓ | Maternal age, education, marital status | Sex, age, activity, birth weight | Pressure to eat 🡺 energy intake (+) |  |  |  | Pressure to eat 🡺 weight (-)  Restriction 🡺 weight (+) | 75 |
|  |  |  |  |  |  |  |  |  |  |  | Indulgent feeding 🡺 energy intake (+) |  |  |  |  |  |
|  |  |  |  |  |  |  |  |  |  |  | Restrictive feeding 🡺 energy intake (-) |  |  |  |  |  |
| Faith et al (2006), USA | WIC eligibility | Hispanic and non-Hispanic | Parents of children aged 1-5 years | 1-5 years | Baseline 1797 | Prospective cohort | Primary Care (WIC) |  | Race/ethnicity, | Sex, race/ethnicity |  |  | Fruit juice consumption in children overweight /at risk of overweight 🡺excess adiposity gain (+) |  | Parent feeding practices | 50 |
|  |  |  |  |  |  |  |  |  |  |  |  |  |  |  | 🡺 weight (ns) |  |
| Welsh et al 2005, USA | WIC eligibility | 88% Black | Parents of children aged 2-3 years | 2-3 years | Baseline 37,421 | Retrospective cohort | Primary Care (WIC) |  |  | Age, sex, race/ethnicity, birth weight, total energy, intake of high-fat foods and sweet foods |  |  | Children overweight at baseline & consumed 1-3 sweet drinks daily 🡺remain overweight (+) |  |  | 75 |
|  |  |  |  |  |  |  |  |  |  |  |  |  |  |  |  |  |
|  |  |  |  |  |  |  |  |  |  |  |  |  | Children at risk of overweight at baseline & consumed 1-3 sweet drinks daily 🡺 become overweight (+) |  |  |  |
|  |  |  |  |  |  |  |  |  |  |  |  |  |  |  |  |  |
|  |  |  |  |  |  |  |  |  |  |  |  |  |  |  |  |  |
|  |  |  |  |  |  |  |  |  |  |  |  |  |  |  |  |  |
|  |  |  |  |  |  |  |  |  |  |  |  |  |  |  |  |  |
| Hoerr et al (2009) USA | Enrolled in Head Start and income at or below 100% of poverty index | African-American, Hispanic American and white | Non-pregnant primary carer, child enrolled in ‘Head start’, <35 years old | <5 years (from Head start website) | 715 | Cross-sectional | Pre-school readiness program (Head Start) |  |  |  | Indulgent or uninvolved parenting compared to authoritarian parenting 🡺 intakes of fruit, juice and vegetables (-) |  |  |  |  | 50 |
|  |  |  |  |  |  |  |  |  |  |  |  |  |  |  |  |  |
|  |  |  |  |  |  |  |  |  |  |  | Indulgent or uninvolved parenting compared to authoritarian parenting 🡺 energy dense evening foods (+) |  |  |  |  |  |
|  |  |  |  |  |  |  |  |  |  |  |  |  |  |  |  |  |
|  |  |  |  |  |  |  |  |  |  |  |  |  |  |  |  |  |
|  |  |  |  |  |  |  |  |  |  |  |  |  |  |  |  |  |
| Hughes et al (2005) USA | Head Start eligibility | African-American and Hispanic | Primary care givers | 3-5 years | 231 | Cross-sectional | Participants in larger study recruited from Head Start | ✓ |  |  |  |  |  |  | Indulgent parenting compared to authoritarian parenting🡺 BMI (+) | 25 |
|  |  |  |  |  |  |  |  |  |  |  |  |  |  |  |  |  |
|  |  |  |  |  |  |  |  |  |  |  |  |  |  |  | Authoritative / uninvolved parenting 🡺 BMI (ns) |  |
|  |  |  |  |  |  |  |  |  |  |  |  |  |  |  |  |  |
|  |  |  |  |  |  |  |  |  |  |  |  |  |  |  |  |  |
|  |  |  |  |  |  |  |  |  |  |  |  |  |  |  |  |  |
| Hurley et al (2013) USA | WIC eligibility or attendance at pediatric clinic serving low income families | Mostly (70%) African-American | Biological mothers | 12–32 months, born at term, birth weight >2500 g | 297 | Cross-sectional | Main study – WIC participants from Toddler Overweight Prevention Study | ✓ |  |  |  |  |  |  | Greater restrictive feeding 🡺toddler overweight (+) | 25 |
|  |  |  |  |  |  |  |  |  |  |  |  |  |  |  |  |  |
|  |  |  |  |  |  |  |  |  |  |  |  |  |  |  | Lower responsive feeding 🡺 toddler overweight (+) |  |
| Murashima et al (2011) USA | Head Start eligibility | Range of ethnicities | >18 years, no dietary conditions like coeliac disease | <5 years | 330 | Cross-sectional | Pre-school readiness program (Head start) |  |  |  | Control / contingency / mealtime behaviour / timing of meals 🡺(ns) |  |  |  | Control / contingency 🡺 BMI (+) | 75 |
|  |  |  |  |  |  |  |  |  |  |  |  |  |  |  |  |  |
|  |  |  |  |  |  |  |  |  |  |  | Child-centred feeding 🡺 nutrient dense food intake (+) |  |  |  |  |  |
|  |  |  |  |  |  |  |  |  |  |  | Encouraging nutrient-dense foods🡺 nutrient dense food intake (+) and energy dense food intake (-) |  |  |  |  |  |
|  |  |  |  |  |  |  |  |  |  |  | Discouraging energy-dense foods 🡺 energy dense food intake (-) |  |  |  |  |  |
|  |  |  |  |  |  |  |  |  |  |  |  |  |  |  |  |  |
|  |  |  |  |  |  |  |  |  |  |  |  |  |  |  |  |  |
| Gibbs Forste (2013) USA | Family SES - composite scale (household income, parental education, occupational prestige) | White (43%), Black (16%), Hispanic (20%), Other (22%) |  | 9 months and 24 months. Nationally representative | Unclear | Prospective longitudinal cohort | Registered births in the National Center for Health Statistics vital statistics system |  | Marital status, race, ethnicity, age at birth, BMI, smoking, depression, use of day care | Number of siblings, gender, birth weight, whether a twin, | Predominant formula feeding 🡺 weight (+) |  | Introduction of solid food before 6 months of age 🡺 weight (+) |  | Predominant formula feeding 🡺 weight (+) | 100 |
|  |  |  |  |  |  |  |  |  |  |  | Early introduction of solid foods 🡺 weight (+) |  |  |  |  |  |
|  |  |  |  |  |  |  |  |  |  |  |  |  | Putting infant to bed with a bottle 🡺 weight (+) |  |  |  |
| Dawson-McClure et al. (2014) USA | Low income communities (based on index of student poverty and minority status) |  |  | Enrolled in pre-k class |  | Pre-post intervention | Public elementary schools |  |  |  |  | Parent knowledge and use of effective parenting practices 🡺 preference for physical activity, time spent walking, engagement in outdoor activities (+), time spent watching TV (-) |  |  | Parent knowledge and use of effective parenting practices 🡺 BMI (ns) | 50 |
| Layte et al. (2014) Ireland | Household social class (occupation) | Irish (86%), UK (2%), Other European Union (7%), African (2%), Other (2%) |  | Nationally representative of children aged 1 year or less | 790 | Longitudinal prospective cohort | Selected from the Child Benefit Register for the Republic of Ireland |  | Maternal nationality, age, weight gain in pregnancy | Sex, birth weight, gestation, birth order, parity, multiple status |  |  |  | Lower levels of TV viewing 🡺 rapid weight gain (-) | Greater duration of breastfeeding and later age of introduction of solids 🡺 weight (+) | 100 |
| Bonuck et al. (2014) USA | WIC eligibility | Hispanic (62%), Non-Hispanic black (16%), Bi- or multiracial (15%), Other (7%) |  | Child consumes >= 2 bottles of milk or juice per day, singleton birth, normal birth weight, no developmental delays | 286 | RCT | Primary care (WIC) |  | Nativity, education level, perception of child's weight | Age, race, gender |  |  |  |  | Frequency of bottle feeding 🡺 weight (ns) Calories consumed via bottle 🡺 weight (ns) | 25 |

1. Indicates whether or not the study reported the use of a theory or conceptual framework. [↑](#footnote-ref-1)
2. + Indicates a statistically significant positive relationship, - indicates a statistically significant negative relationship, ns indicates no statistically significant relationship [↑](#footnote-ref-2)
3. MMAT (Mixed Methods Appraisal Tool) quality rating score from 25% (poor) to 100% (good) [↑](#footnote-ref-3)
4. Special Supplemental Nutrition Program for Women, Infants and Children [↑](#footnote-ref-4)
5. Randomised Controlled Trial [↑](#footnote-ref-5)
6. Sugar-sweetened beverage [↑](#footnote-ref-6)
